# Lung cancer vaccine strategies: exploring the spectrum from traditional to RNA-based platforms

**DOI:** 10.3389/fbioe.2025.1617352

**Published:** 2025-06-23

**Authors:** Alireza Pazoki, Sepehr Dadfar, Atefe Alirezaee, Valentyn Oksenych, Dariush Haghmorad

**Affiliations:** ^1^ Department of Immunology, School of Medicine, Semnan University of Medical Sciences, Semnan, Iran; ^2^ Student Research Committee, Semnan University of Medical Sciences, Semnan, Iran; ^3^ Immunology, Asthma and Allergy Research Institute, Tehran University of Medical Sciences, Tehran, Iran; ^4^ Pediatrics Center of Excellence, Children’s Medical Center Hospital, Tehran University of Medical Sciences, Tehran, Iran; ^5^ Broegelmann Research Laboratory, Department of Clinical Science, University of Bergen, Bergen, Norway

**Keywords:** lung cancer, immunotherapy, cancer vaccines, therapeutic vaccines, cancer therapy

## Abstract

Lung cancer continues to be a leading cause of cancer-related mortality worldwide, with survival rates stubbornly low despite significant advancements in conventional therapies. The limited effectiveness of traditional immunotherapies, particularly in advanced stages of the disease, highlights an urgent need for innovative treatment options. Cancer vaccines represent a promising Frontier in this battle, aiming to harness the power of the immune system to create lasting memory against tumor cells. This approach not only promotes tumor regression but also does so with minimal adverse effects. The death of tumor cells induced by these vaccines triggers a cascade of immune responses, activating functional T cells and sustaining the cancer-immunity cycle. Among emerging platforms, RNA-based vaccines have garnered particular attention for their rapid development potential, flexible design, and ability to induce robust cellular and humoral immunity. As a result, cancer vaccines—including RNA-based modalities—are increasingly viewed as a groundbreaking therapeutic strategy in the immunotherapy landscape for solid tumors. In this review, we examine recent advancements in lung cancer vaccines, focusing on antigen selection, innovative vaccine platforms and delivery strategies. Moreover, we provide a detailed analysis of ongoing and completed clinical trials, including targeted antigens, administration routes, and platforms used. Additionally, we discuss the potential benefits of combination therapies to enhance vaccine efficacy and address the limitations of these vaccines. Our goal is to provide a comprehensive overview of how these developments aim to overcome current treatment challenges and improve patient outcomes.

## 1 Introduction

Cancer remains a significant global health concern and is the leading cause of death worldwide. Among all types of cancer, lung cancer was the most frequently diagnosed and the leading cause of cancer-related mortality, accounting for an estimated 1.8 million deaths (Bray et al., 2024; Karankar et al., 2025). The World Health Organization (WHO) categorizes lung tumors into two major groups: non-small cell lung cancer (NSCLC), which represents 80%–85% of all cases, and small cell lung cancer (SCLC), accounting for the remaining 15% (Travis et al., 2015). Despite advances in conventional treatments such as surgery, chemotherapy, and radiotherapy, the survival rates for metastatic lung cancer, including both NSCLC and SCLC, remain alarmingly low, with a 5-year rate of approximately 4% (Siegel et al., 2022; Boloker et al., 2018).

**FIGURE 1:**
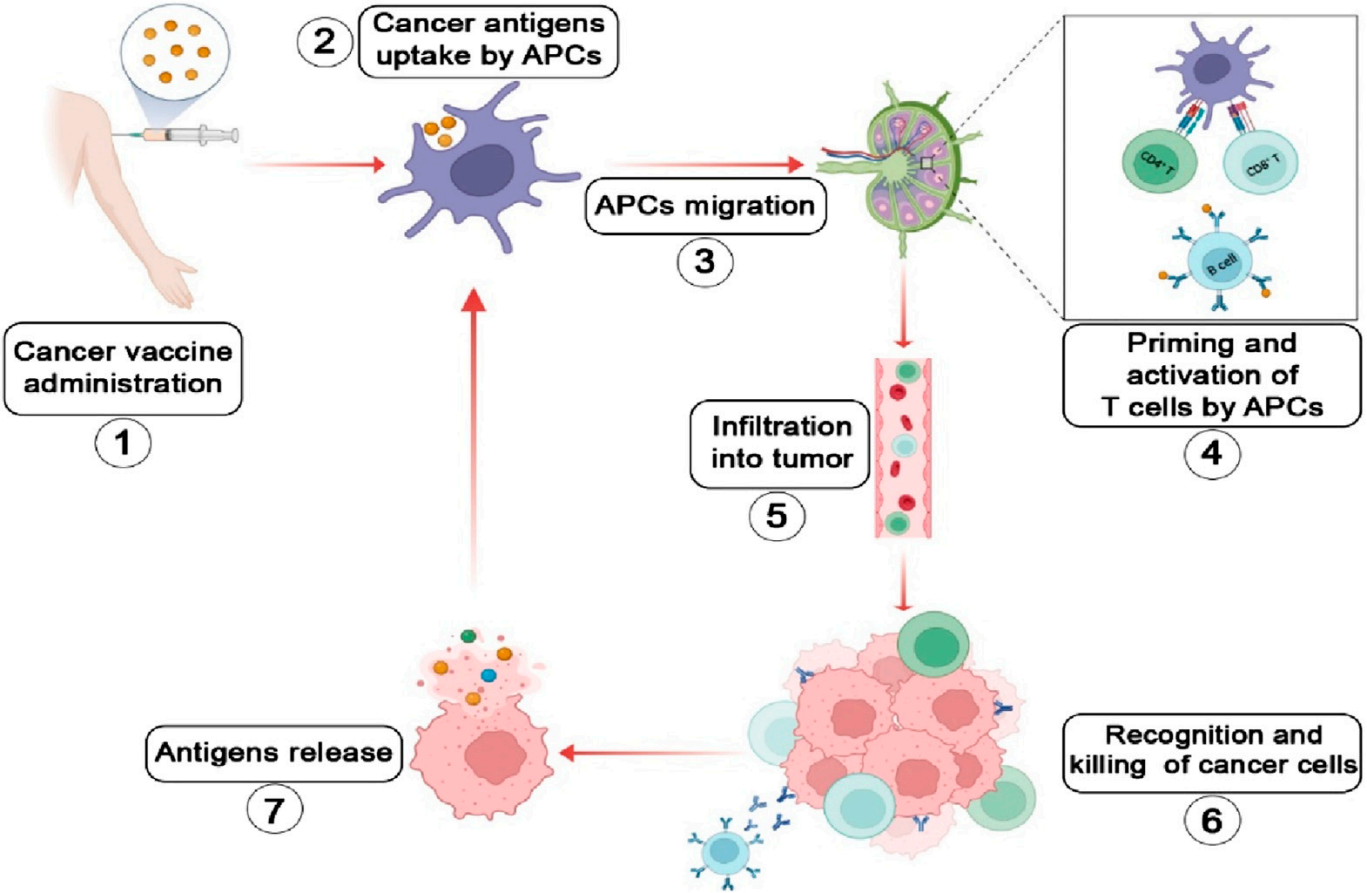
Schematic illustration of the tumor-immune cycle induced by cancer vaccines (Bray et al., 2024): Cancer vaccine immunization (Karankar et al., 2025); Antigens are phagocytosed, expressed intracellularly, and efficiently processed by dendritic cells (DCs) (Travis et al., 2015); Antigen-loaded DCs migrate to lymph nodes (Siegel et al., 2022); DCs present antigens on MHC class I (*via* cross-presentation) and class II molecules to CD8^+^ and CD4^+^ T cells, respectively. Activated T cells undergo proliferation and differentiate into memory T cells and effector T cells. Follicular dendritic cells facilitate the development of memory B cells and plasma cells (Boloker et al., 2018); The activated and expanded T cells infiltrate the tumor microenvironment (Ruzzi et al., 2024); Effector T cells directly eliminate tumor cells or induce their apoptosis. Activated B cells promote tumor apoptosis through antibody-dependent cellular cytotoxicity (ADCC) (Lei W. et al., 2025); The killing of tumor cells releases more antigens, boosting the diversity and breadth of the immune response (Sautes-Fridman et al., 2019). The Figure is designed using BioRender.com.

Cancer vaccines are a type of immunotherapy designed to eliminate tumor cells primarily by stimulating cellular immunity and initiating the cancer-immunity cycle, thereby providing a sustained anti-tumor effect (Figure 1) (Ruzzi et al., 2024). Cancer vaccines can be used in both prophylactic and therapeutic settings. Prophylactic vaccines are designed to prevent infections caused by oncogenic viruses. To date, the HBV and HPV vaccines for liver and cervical cancers are the only preventive cancer vaccines that have successfully completed clinical trials and received FDA approval (Lei W. et al., 2025). For other malignancies, such as lung cancer, which is not primarily caused by viral infections, vaccines typically serve a therapeutic role by stimulating antitumor immunity. Recently, LungVax, an experimental vaccine developed by researchers at the University of Oxford, the Francis Crick Institute, and University College London, has been designed to prevent NSCLC in high-risk populations, including smokers and former smokers.

In contrast to prophylactic vaccines, therapeutic vaccines focus on enhancing the immune system’s ability to eliminate cancer cells, primarily targeting tumor-specific antigens. Most cancer vaccines currently under investigation are therapeutic rather than preventive.

This review outlines the principal antigenic targets and vaccine platforms investigated for lung cancer, with particular emphasis on mRNA-based approaches. It further summarizes representative clinical trials across different platforms, providing insight into current advancements and ongoing challenges in the development of effective lung cancer vaccines.

## 2 Cancer vaccine antigens

**FIGURE 2:**
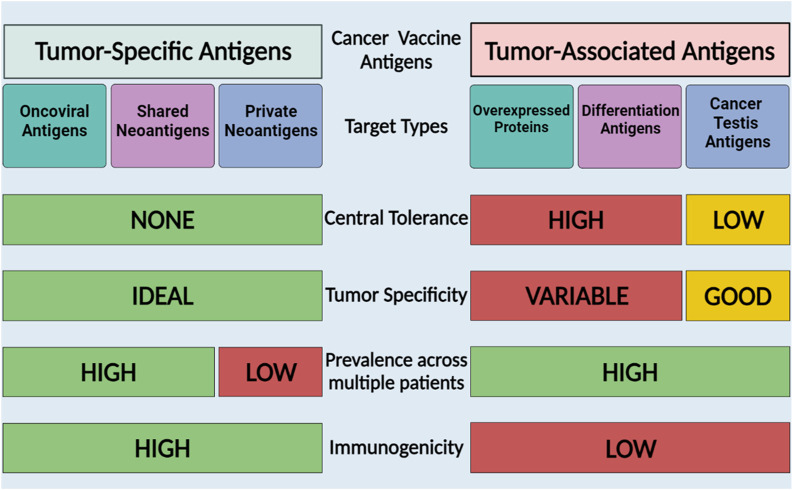
Cancer vaccine antigen types and characteristics. TAAs are self-proteins expressed in cancer cells and TSAs are proteins expressed by tumor cells that may arise from mutations or viruses. The Figure is designed using BioRender.com.

One of the key steps in the development of a cancer vaccine is the selection of an appropriate antigen. The effectiveness of cancer vaccines relies on the recognition of tumor antigens by T cells (Coulie et al., 2014). Tumor antigens can be classified into two primary categories: tumor-associated antigens (TAAs), which are self-antigens abnormally expressed in cancer cells and are also found in normal cells, and tumor-specific antigens (TSAs) which are produced by tumor-specific somatic mutations and are exclusively expressed by cancerous cells (Motz and Coukos, 2013). Figure 2 summarizes the characteristics of TAAs and TSAs, moreover, Tables 1–4 summarizes some of the clinical trials involving TSAs and TAAs in lung cancer.

**TABLE 1:** Summary of selected Cell-based lung cancer vaccination clinical trials listed on ClinicalTrials.gov.

NCT	Phase	Enrollment	Status	Vaccine component	Antigen type	Cancer type	ROA	Components
NCT05886439 (LK101)	I	40	Recruiting	personalized neoantigen pulsed DC vaccine	TSA	NSCLC, SCLC	N/A	LK101 injection with Pembrolizumab or Durvalumab
NCT03546361 (Ad-CCL21-DC)	I	24	Active	CCL21-gene-modified dendritic cell vaccine	TAA	NSCLC	IT	Intratumoral gene-modified dendritic cell vaccine (CCL21) alongside intravenous pembrolizumab
NCT03970746 (PDC*lung01)	I/II	73	Active	NY-ESO-1 MAGE-A3 MAGE-A4 Multi-MAGE Survivn MUC1 Melan-A	TAA	NSCLC	SC	Irradiated human plasmacytoid dendritic cells (PDC) loaded with seven synthetic peptides from lung tumor antigens
NCT03205930 (Neo-MASCT)	I/II	20	Unknown	Neoantigen pulsed DC and CTL cells vaccine	TSA	NSCLC	SC	Combination of a dendritic cell (DC) vaccine with neoantigen-specific T cells
NCT02470468 (DCVAC/LuCa)	I/II	105	Completed	Tumour antigens DC vaccine	TAA	NSCLC	SC	DCVAC/LuCa combined with carboplatin and paclitaxel, with or without immune enhancers (interferon-α and hydroxychloroquine)
NCT01782287	II/III	60	Unknown	Tumour specific antigen DC vaccine	TSA	BMLC	SC	dendritic cell vaccine
NCT01829373	I	11	Completed	Tumour associated antigen DC vaccine	TAA	LC	ID	Cellular vaccine composed of killed allogeneic tumor cells (1650-G) + beta glucan + GM-CSF.
NCT00676507 (belagenpumatucel-L)	III	532	Completed	TGF-beta2 antisense gene-modified allogeneic tumor cell vaccine.	TAA	NSCLC	ID	Allogeneic NSCLC cells with a plasmid containing a TGF-beta2 antisense transgene
NCT00654030	II	12	Completed	Allogeneic tumor cell vaccine	TAA	NSCLC	ID	A pluripotent, allogeneic, tumor cell vaccine composed of irradiated tumor cells from the NSCLC cell line 1650 and the immunoadjuvant recombinant GM-CSF
NCT00089726	II	100	Completed	Atulogus tumor cell vaccine	TAA	NSCLC	ID	GM-CSF gene-modified autologous tumor vaccine (CG8123), with and without low-dose cyclophosphamide
NCT01058785 (Lucanix™)	II	75	Completed	Allogeneic tumor cell vaccine	TAA	NSCLC	ID	TGF-beta2 antisense gene-modified allogeneic tumor cell vaccine

Abbreviations: NCT, national clinical trial; ROA, route of administration; DC, dendritic cell; TSA, Tumor-Specific Antigen; TAA, Tumor-Associated Antigen; NSCLC, Non-Small Cell Lung Cancer; SCLC, small cell lung cancer; N/A, not applicable; SC, subcutaneous; CCL, Chemokine (C-C motif) Ligand; NY-ESO, New York esophageal squamous cell carcinoma-1; MAGE, melanoma antigen family; CTL, Cytotoxic T Lymphocyte; GM-CSF, Granulocyte-Macrophage Colony-Stimulating Factor; MUC1:Mucin 1; BMLC, brain metastases lung cancer; ID, intradermal; IT, intratumoral; TGF, transforming growth factor.

### 2.1 Tumor-associated antigens (TAA)

TAAs, also known as tumor-shared antigens, are classified into several categories, including cancer/testis antigens (CTAs) typically found in immune-privileged germline cells, cell lineage differentiation antigens usually absent in adult tissues, and antigens that are overexpressed in cancer cells (Hollingsworth and Jansen, 2019). Antigens such as Melanoma-associated antigen A1 (MAGE-A1), MAGE-A3, Mucin 1 (MUC1), New York esophageal squamous cell carcinoma 1 (NY-ESO-1) and Epidermal growth factor receptor (EGFR) are some of the main TAAs that use in clinical trials in lung cancer (Lahiri et al., 2023; Alenezi, 2025).

NY-ESO-1, a highly immunogenic molecule, is typically expressed in germ and placental cells but is re-expressed in various cancers, including NSCLC (Thomas et al., 2018). MAGE-A1 was the first CTA identified as being significantly expressed in melanoma and NSCLC. Additionally, MAGE-A1-specific CD8^+^ and CD4^+^ T lymphocytes have been observed in NSCLC patients (Alsalloum et al., 2023). MAGE-A3 is another CTA, with studies reporting that 35% of NSCLC cases express MAGE-A3. Its expression is positively correlated with disease stage, reaching 50% in stage II (Sienel et al., 2004). Numerous epithelial adenocarcinomas, including those of the lung, liver, colon, breast, pancreas, and ovaries, commonly overexpress MUC1. MUC1 is the second-best potential TAA for creating cancer vaccines, according to the National Cancer Institute (Cheever et al., 2009). EGFR was the first oncogenic target identified in NSCLC, found in over 60% of patients. Kinase-activating mutations result in elevated tyrosine kinase activity and are commonly observed in NSCLC and glioblastoma (Karlsen et al., 2021).

Many clinical trials targeting TAAs with vaccines have shown detectable immune responses, but these responses often lack the strength needed to produce significant clinical efficacy. For instance, phase III trials of TAA-targeting vaccines in NSCLC (MAGE-A3, MUC-1) have not shown positive outcomes to date (Jou et al., 2021). Additionally, since TAAs are also expressed in normal tissues, there is an increased risk of vaccine-induced autoimmune toxicity (Gianneschi et al., 2025).

### 2.2 Tumor-specific antigens (TSA)

TSAs are proteins expressed by tumor cells that can result from mutations (neoantigens) or viruses engaged in oncogenic transformation (oncoviral antigens). Neoantigens can be divided into two types: shared neoantigens and personalized neoantigens (Tureci et al., 2018; Schumacher and Schreiber, 2015). Shared neoantigens with high immunogenicity have the potential to be used as broad-spectrum therapeutic cancer vaccines for patients with the same mutant gene (Klebanoff and Wolchok, 2018; Zhao et al., 2020). Personalized neoantigens are mutated antigens unique to each individual and vary significantly from patient to patient. As a result, drugs targeting personalized neoantigens must be tailored specifically to each individual, representing a form of personalized therapy (Tureci et al., 2018). Oncoviral antigens are proteins produced by viruses that drive oncogenic transformation. Since oncogenic viruses are common across certain types of tumors, this class of antigens is not specific to individual patients (Hollingsworth and Jansen, 2019; Vigneron, 2015).

There are several potential advantages to using tumor vaccines composed of TSAs rather than TAAs. Neoantigens are believed to be highly immunogenic, as well as, since tumor neoantigens are only expressed by tumor tissue, targeting them prevents T lymphocytes from destroying healthy tissue (Hernandez and Malek, 2022). However, not all neoantigens are immunogenic, and specific criteria must be met for a neoantigen to trigger anti-tumor immunity. These include sufficient production of the neoantigen, strong binding affinity to the patient’s HLA molecules, and effective recognition by the patient’s T cells (Harndahl et al., 2012). Furthermore, tumors may experience antigen loss, which makes antigen-specific immune responses against the lost antigen ineffective (Ott et al., 2017).

## 3 Cancer vaccines platforms

**FIGURE 3:**
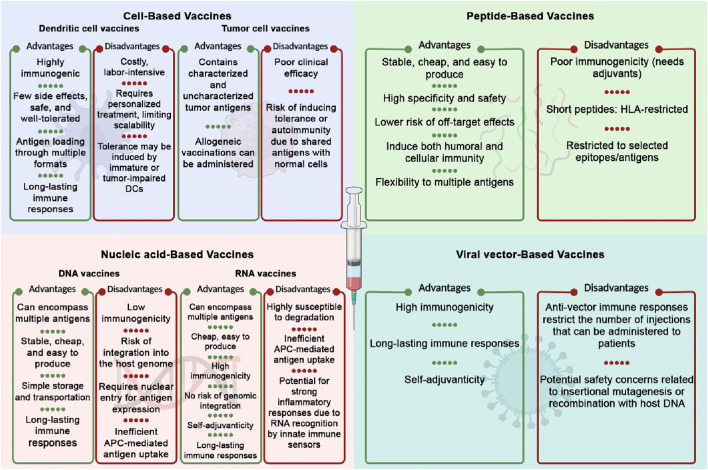
Advantages and disadvantages of different cancer vaccine platforms. The Figure is designed using BioRender.com.

The success of a cancer vaccine in inducing an immune response relies heavily on selecting the appropriate platform or delivery strategy. Cancer vaccines can be classified into four categories: cell-based vaccines, peptide-based vaccines, viral-based vaccines, and nucleic acid-based vaccines (Kamel et al., 2025). The advantages and disadvantages of each category are summarized in Figure 3.

### 3.1 Cell-based cancer vaccines

Cell-based vaccines can be developed using either autologous or allogeneic tumor cells. Allogeneic vaccinations have the benefit of reducing time even though they are not personalized. In contrast, autologous vaccines use the patient’s own tumor cells, ensuring better antigen compatibility, but they come with higher costs and longer preparation times. Cellular vaccines may utilize whole tumor cell lysates or antigen-loaded autologous antigen-presenting cells, most commonly dendritic cells, or a combination of both (Le et al., 2010). The whole tumor cell (WTC) vaccine represents a straightforward and direct method for tumor immunotherapy. These vaccines have the ability to stimulate a wide range of immune cells, such as T cells, B cells, and NK cells (Liu D. et al., 2023). Live tumor cells are poorly immunogenic in part because they actively secrete various immunosuppressive soluble factors that inhibit the function of DCs and T cells (Chiang et al., 2010). For instance, tumor-derived vascular endothelial growth factor (VEGF) suppresses DC differentiation and maturation, while soluble Fas ligand can induce apoptosis in activated lymphocytes. Additionally, tumors release soluble MICA, which impairs NKG2D-mediated killing by immune cells. Other immunosuppressive molecules include IL-10 and TGF-β, both of which dampen antigen presentation and T cell responses. Furthermore, galectin-1 and indoleamine 2,3-dioxygenase (IDO) inhibit T cell proliferation and activation. These factors collectively create a highly immunosuppressive tumor microenvironment that hampers the effectiveness of whole tumor cell vaccines (Chiang et al., 2010).

Therefore, several strategies are employed to increase the immunogenicity of these cells. For instance, immunological responses to dead cells are more potent than those from living cells (Gamrekelashvili et al., 2015). Additionally, modifications to tumor cells can enhance antigen presentation, a critical factor in improving vaccine efficacy. For instance, studies have shown that WTC vaccines genetically engineered to express IL-21 and IL-7 exhibit high therapeutic effectiveness. (Gu et al., 2016). In addition, various adjuvant decoration procedures have been employed to enhance the vaccination. For example, dying tumor cells decorated by CpG-loaded nanoparticles were reported to improve antigen presentation (Fan et al., 2017).

Incorporating tumor-associated antigens into DCs is an effective method for enhancing tumor immunity. This strategy typically includes the *ex vivo* generation or isolation of autologous dendritic cells (DCs) from a patient’s cytapheresis, either from circulating or monocyte-derived cells. The DCs are then matured, primed with antigens (such as mRNA, DNA, peptides, or tumor cell lysates), and reinfused into the patient (Sabado et al., 2017).

For DC vaccination, monocyte-derived DCs (moDCs) have been widely used due to their ability to be easily differentiated *ex vivo* from monocytes, which can be collected in large quantities through leukapheresis. However, recent perspectives suggest that moDCs may not be the most optimal DC subtype for vaccination (Bol et al., 2019; Bol et al., 2016). Monocyte-derived dendritic cells (moDCs), which arise from monocytes during inflammation rather than from conventional DC precursors, display limited cross-presentation capacity and reduced expression of key costimulatory molecules compared to other dendritic cell subsets, such as cDC1s. This is attributed to their distinct transcriptional programming, lack of specialized antigen-processing machinery (e.g., WDFY4, SEC22B), and the immunosuppressive influence of the tumor microenvironment, including factors like IL-10, PGE2, and lipid accumulation that impair their maturation and function. Consequently, moDCs are less effective in priming CD8^+^ T cell responses and may instead contribute to immune regulation or tolerance in cancer settings (Wculek et al., 2020). In addition, moDCs have limitations with their generation time and limited functionality. On the other hand, because naturally occurring DCs express higher MHC molecules, they are better able to deliver antigens (Wculek et al., 2020). Thus, a key challenge for the future development of DC vaccines is optimizing *in vitro* culture conditions to generate high-quality DC subsets. To address this challenge, several optimization strategies have been proposed. Modifying cytokine combinations used during DC differentiation, such as supplementing or replacing GM-CSF and IL-4 with IL-15 or IFN-α, can enhance DC immunogenicity and improve their capacity to prime T cells (Palucka and Banchereau, 2013). Additionally, employing three-dimensional (3D) culture systems that better mimic physiological conditions may promote the generation of more functional and clinically relevant DC subsets (Wculek et al., 2020). The use of small molecules or metabolic modulators that target pathways like mTOR and β-catenin has also shown promise in reprogramming DC functionality, leading to improved antigen presentation and cytokine production (Vander et al., 2014). Moreover, replacing GM-CSF with FLT3 ligand supports the differentiation of a broader range of conventional DCs, including the highly effective cross-presenting cDC1 subset (Guilliams et al., 2016). These approaches collectively aim to produce DCs with enhanced expression of MHC molecules and costimulatory markers, and improved capacity to induce robust anti-tumor immune responses. Moreover, the transportation and storage of cell-based vaccines under strict conditions pose logistical challenges, which could compromise their viability during distribution.

### 3.2 Peptide-based cancer vaccines

Peptide-based vaccines are composed of polypeptides that include known or predicted tumor antigen epitopes. (Lei Y. et al., 2025). Peptide-based vaccines typically have low immunogenicity because of the limitations of MHC polymorphism and the small size of antigen epitopes.

The effectiveness of the peptide vaccination is mostly determined by the length of the peptide chain. Short peptides, typically consisting of fewer than 15 amino acids, are processed intracellularly and loaded onto MHC class I molecules *via* the endogenous antigen presentation pathway within nucleated cells (Rock et al., 2016). However, when short peptides are administered alone, they can be presented by non-professional antigen-presenting cells (APCs) that lack the required costimulatory signals, potentially inducing T cell anergy or tolerance (Hailemichael et al., 2013; Toes et al., 1996; Bijker et al., 2008). Long peptides, as opposed to short peptides, enable greater coverage of HLA with many epitopes, facilitate motif recognition and binding, and boost immunogenicity (Southwood et al., 1998). Unlike short peptides, long peptides need to be processed by APCs before they can be presented on MHC molecules. Once internalized, a portion of the long peptides is degraded through the endosomal pathway, loaded onto MHC class II molecules, and recognized by CD4^+^ T helper cells. The remaining portions enter the cytoplasmic or vacuolar pathway and are cross-presented by MHC class I molecules to activate CD8^+^ T cells (Jhunjhunwala et al., 2021). As a result, long peptide vaccines have a greater potential to elicit durable and robust anti-tumor immune responses.

Vaccines against synthetic long peptides (SLPs) usually comprise 25–35 amino acids, often covering many epitopes or greater sections of the target protein (Bijker et al., 2008). Therefore, by employing longer peptide sequences, SLP vaccines with numerous epitopes can elicit larger and more broadened immune responses. Peptide stability and antigen delivery effectiveness can be further improved by using SLPs as opposed to short peptides (Chen et al., 2020). However, SLP cancer vaccines have drawbacks, including complicated preparation, the potential for HLA restriction, and rapid degradation. The preparation of synthetic long peptides is much more complex than that of short peptides due to increased risk of aggregation, solubility issues, cumulative yield loss, and the need for advanced synthesis and purification strategies. Specialized methods such as fragment condensation and segmental synthesis are often required for long peptides, making their production more technically demanding, time-consuming, and costly compared to short peptides (Shah et al., 2025). Thus, it is essential to develop more effective immune formulations to enhance peptide-specific immunity.

### 3.3 Viral vector-based cancer vaccines

Virus-based vaccines can be categorized into three types: inactivated, live attenuated, or subunit vaccines targeting the virus that may lead to tumor formation; oncolytic virus vaccines; and virus vector vaccines.

Viral vectors or virus-like particle-based vaccines have been extensively studied as vector platforms because of their intrinsically immunogenic character and the capacity to efficiently insert genetic material into cells (Sasso et al., 2020). Poxviruses, adenoviruses, and alphaviruses are the most often used viral vaccine vectors; for safety, replication-defective or attenuated strains are favored (Larocca and Schlom, 2011). A key advantage of virus-based vaccines is that the immune system has evolved to respond to viruses effectively, with both innate and adaptive mechanisms working together to produce an intense and long-lasting response. Oncolytic virotherapy is a promising approach for enhancing the efficacy of cancer vaccines by modulating the TME and selectively targeting and killing malignant tissue while sparing normal cells and surrounding tissues (Wang et al., 2025). When tumor cells are infected by an oncolytic virus, they generate reactive oxygen species (ROS) and cytokines, which activate immune cells. This process leads to oncolysis, releasing a variety of immunogenic substances, including viral proteins, nucleic acids, TAAs, PAMPs, DAMPs, and immunogenic neoepitopes (Kaufman et al., 2015). The effectiveness of oncolytic viruses against tumors has been demonstrated in numerous clinical trials, with T-VEC, a first-generation recombinant herpes simplex virus, emerging as the most notable example to date (Russell and Barber, 2018).

### 3.4 Nucleic acid-based cancer vaccines

Nucleic acid-based vaccines stimulate the host immune system by delivering the coding region of an antigen through DNA or RNA, resulting in the production of specific antigens (Pallerla et al., 2021; Liao and Liu, 2025). These vaccinations offer a number of benefits. (Bray et al., 2024). They can encode full-length tumor antigens, allowing for the presentation of multiple epitopes *via* MHC class I and II pathways, thereby generating a broader and more robust CD4^+^ and CD8^+^ T cell response (Sahin et al., 2014). (Karankar et al., 2025) Nucleic acid vaccination can enhance the production of pro-inflammatory cytokines (such as IFN-α, IL-6, and TNF-α) and stimulate pattern recognition receptors (e.g., TLR3, TLR7/8, STING), which leads to the maturation and activation of dendritic cells (Pardi et al., 2018). (Travis et al., 2015) Fusion gene strategies can be employed to co-express helper epitopes or cytokines (e.g., GM-CSF, IL-12) along with tumor antigens, thereby promoting T-helper memory cell differentiation and sustaining long-term immune responses (van der Burg et al., 2016). These features, along with their safety, scalability, and adaptability, position nucleic acid-based vaccines as promising tools in cancer immunotherapy. Gene-based vaccinations are divided into two categories: DNA-based vaccines and RNA-based vaccines.

Adjuvants are not as necessary for nucleic acid-based vaccines because they can also trigger nucleic acid sensors that activate DCs, including certain TLRs, STING, AIM2, and DAI pathways. Furthermore, because nucleic acid vaccines do not strongly elicit anti-vector immunity, they can be dosed repeatedly (Jorritsma et al., 2016).

#### 3.4.1 DNA vaccine

Cancer DNA vaccines are based on bacterial plasmids that encode one or more cancer antigens, which activate both innate and adaptive immune responses (Karaliota et al., 2025). These plasmids can also encode immunostimulatory cytokines, such as GM-CSF and IL-2 (Liu, 2011). DNA is more stable and has a longer half-life in the body than mRNA due to the widespread presence of RNA-degrading enzymes and structural differences between DNA and RNA. As a result, DNA vaccines were the primary focus of early nucleic acid vaccine development (Nigar and Shimosato, 2019; Roy et al., 2020). However, DNA vaccines may cause insertion mutations, whereas mRNA vaccines do not. There is a theoretical concern that DNA vaccines could cause insertional mutagenesis if the plasmid DNA (pDNA) integrates into the host genome. Although rare, such integration events could potentially disrupt essential genes, including tumor suppressors or activate oncogenes, leading to unintended genomic alterations. However, this risk remains largely hypothetical and has not been observed in clinical trials to date (Pagliari et al., 2023). Despite years of research into DNA vaccines, progress has been limited. Nonetheless, India recently approved a COVID-19 DNA vaccine, ZycoV-D, marking it as the world’s first DNA vaccine for human use (Khobragade et al., 2022). This approval may pave the way for DNA vaccines to be applied to a broader range of diseases.

The DNA must enter the nucleus, where it is transcribed and translated into antigens in the cytoplasm. A single plasmid DNA can generate several mRNA copies once it enters the nucleus, producing more antigens than a single mRNA molecule. CpG motifs in plasmid DNA can stimulate innate immune responses by acting as danger signals that interact with Toll-like receptor 9 (TLR9). When TLR9 is engaged, it initiates a signaling cascade that activates NF-κB and IRAK, leading to the production of chemokines and inflammatory cytokines (Nigar and Shimosato, 2019). The double-stranded structure of DNA also activates the STING signaling pathway (Ishikawa et al., 2009).

However, due to their low immunogenicity, DNA vaccines have only made restricted advances in clinical studies (Suschak et al., 2017). There are numerous methods for enhancing DNA vaccine immunogenicity. Plasmid element optimization is one of the key approaches. For example, species-specific codons, the Kozak sequence prior to the initiation codon, and the intron sequence should be taken into account (Saade and Petrovsky, 2012). In addition, effective transcription requires a strong promoter sequence. To increase efficacy, DNA vaccines are often combined with various methods and adjuvants, such as cytokines, immune checkpoint inhibition, chemotherapy, radiation, and endocrine therapy (Lopes et al., 2019).

#### 3.4.2 RNA vaccine

Although the topic of RNA vaccines for cancer is relatively new, RNA has long been investigated for medicinal purposes. Despite initial difficulties in achieving stability, immunogenicity, and effective delivery, the emergency use of two mRNA COVID-19 vaccines has once again drawn attention to the development of RNA vaccines.

mRNA vaccines provide several advantages, including high potency, safe administration, rapid development potential, and cost-effective manufacturing (Miao et al., 2021; Chehelgerdi and Chehelgerdi, 2023). Moreover, multiple antigens and full-length tumor antigens can be encoded simultaneously using mRNA vaccines. Encoding many antigens promotes broader humoral and cellular immunity, increasing the possibility of overcoming cancer vaccine resistance (Van Nuffel et al., 2012). Consequently, mRNA provides a perfect platform for creating personalized neoantigen vaccines (Pardi et al., 2018; Fu et al., 2025). Since mRNA is created by *In Vitro* Transcription (IVT) and can be translated directly into protein once it enters the cytoplasm, it provides a well-tolerated delivery mechanism without the risk of genome integration, as opposed to DNA vaccines (He et al., 2022). IVT transcribes mRNA *in vitro* using a linearized DNA template and bacteriophage RNA polymerase. This cell-free process simplifies, accelerates, and clarifies mRNA production by avoiding complex cellular regulations (Pardi et al., 2020). Although RNA is more prone to degradation by common RNases, this vulnerability can be reduced through chemical modifications and the inclusion of modified nucleosides like pseudouridine (Kariko et al., 2008). The mRNA is also transiently produced in cells, allowing for repeated immunizations (Pardi et al., 2018).

Self-amplifying RNA (SAM) and non-replicating mRNA are the two primary categories of mRNA vaccines. However, cancer mRNA vaccines are typically non-replicating. Non-replicating mRNA consists of a 7-methylguanosine (m7G) 5′ cap, a 5′-untranslated region (5′-UTR), an open reading frame (ORF), a 3′-untranslated region (3′-UTR), and a 3′ poly(A) tail (Pardi et al., 2018). These elements are critical for mRNA stability and transcription factor recruitment, which influence protein translation efficiency. SAM is derived from alphavirus and is capable of replicating and multiplying *in vivo* to elicit a lasting and effective immune response. SAM enables the production of large quantities of antigen from small vaccine doses over an extended period. Nevertheless, the utilization of SAM in cancer vaccines is currently limited to preclinical research, and additional investigation is required before its clinical implementation can be considered. Unlike non-replicating mRNA, SAM contains two ORFs: one that encodes the target antigen and another that encodes the viral replication machinery, which facilitates prolonged RNA amplification within cells (Bloom et al., 2021).

The main challenges in developing mRNA vaccines revolve around their molecular design and the efficiency of their delivery *in vivo*. Several mRNA delivery strategies have been developed to extend the mRNA circulation period *in vivo*, enhance translation efficiency, and boost antigen uptake by APCs while reducing the extracellular destruction of naked mRNA by RNA enzymes.

Stabilizing mRNA is essential for ensuring its effective expression. There are various approaches that can be taken to enhance mRNA stability and translation efficiency. The 5′ cap is vital for efficient translation of mRNA into protein (Shuman, 2002), while the poly(A) tail further enhances translation efficiency by stabilizing the mRNA and reducing exonuclease-mediated degradation (Mangus et al., 2003). Additionally, optimizing untranslated regions (UTRs) improves both mRNA stability and translation efficiency through interactions with various transcription factors (Miao et al., 2021; Pickering and Willis, 2005). Furthermore, improved translation efficiency is achieved by avoiding hairpin loops, substituting rare codons, and maintaining an appropriate GC content (Linares-Fernandez et al., 2020).

Innate immune activation is another important barrier impeding the development of mRNA vaccines. Through a variety of RNA sensors, including TLRs, RIG-I, and PKR, mRNA triggers the innate immune response (Nallagatla et al., 2007; Rehwinkel et al., 2010; Heil et al., 2004; Alexopoulou et al., 2001). While this immune activation can act as an adjuvant and enhance vaccine effectiveness, it can also inhibit mRNA translation. To mitigate innate immune activation, mRNA transcripts can be modified by substituting nucleotides, such as replacing cytidine with 5-methylcytidine (m5C) or uridine with pseudouridine (Ψ) or 1-methylpseudouridine (m1Ψ) (Weng et al., 2020). Additionally, impurities like double-stranded RNA (dsRNA) in mRNA can activate pattern recognition receptors (PRRs). High-purity mRNA, achieved through purification techniques like high-performance liquid chromatography (HPLC), minimizes unwanted immune activation by removing these contaminants (Weissman et al., 2013).

## 4 Delivery Systems for Cancer Vaccines

**FIGURE 4:**
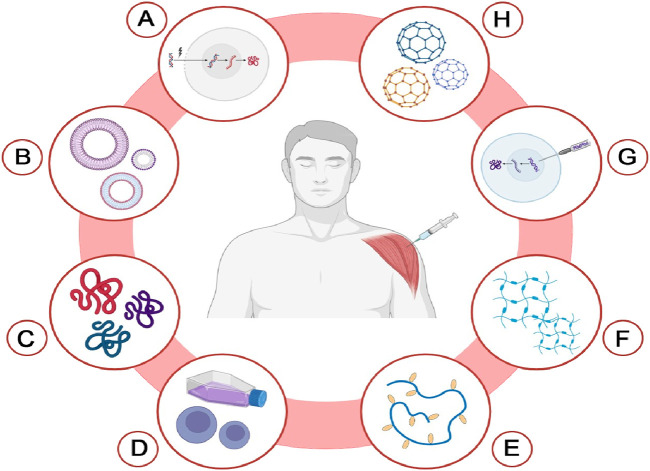
Delivery systems for cancer vaccines. This diagram illustrates various innovative delivery systems utilized for cancer vaccines. Each method is represented as follows: **(A)** Electroporation-based delivery; **(B)** Extracellular vesicle (EV)-based delivery; **(C)** Self assembling peptide-based delivery; **(D)** Cell-based delivery; **(E)** Cholesteryl group-modified pullulan (CHP)-based delivery; **(F)** Hydrogel-based delivery; **(G)** Gene gun-based delivery; **(H)** Nanoparticle-based delivery. The Figure is designed using BioRender.com.

Significant limitations, including low efficacy, side effects, poor tumor penetration, and increased toxicity, often hinder the effectiveness of immunotherapy and lung cancer vaccines (Lahiri et al., 2023). However, these challenges can be effectively mitigated through the use of advanced delivery systems (Kudling et al., 2022; Li et al., 2022; Rosenblum et al., 2018; Yao et al., 2022). An optimal delivery system enhances the targeted delivery of vaccines and facilitates the concurrent administration of other therapeutic agents, enabling a combined therapeutic approach. In this section, we will explore commonly used delivery systems for administering cancer vaccines, with a focus on those designed for lung cancer. Figure 4 illustrates the different delivery systems used for cancer vaccines.

### 4.1 Nanoparticle-based delivery

Nanoparticles can deliver antibodies, peptides, proteins, and small molecules (Chen, 2010; Xu et al., 2019). This method represents an effective approach for administering vaccines that previously faced pharmacokinetic challenges, such as low bioavailability (Wen et al., 2016). Several types of nanoparticles have been investigated for cancer vaccination, including liposomes, inorganic nanocarriers, dendrimers, polymeric systems, nucleic acid nanotechnology, micelles, carbon nanotubes, mesoporous silica nanoparticles, and gold nanoparticles. These can be utilized alone or in combination to enhance therapeutic efficacy (Wen et al., 2016; George et al., 2022).

Among these, liposomes are particularly common as nanoparticle-based vaccine carriers. By adjusting factors such as lipid composition, surface charge, particle size, and surface modifications (e.g., PEGylation), researchers can tailor liposomes to meet specific therapeutic and immunological requirements. These include prolonging circulation time by avoiding rapid clearance through the reticuloendothelial system, enhancing tumor accumulation *via* the enhanced permeability and retention (EPR) effect, improving uptake by APCs, and controlling the release rate of encapsulated antigens or adjuvants. For example, incorporating cholesterol can improve membrane stability, while cationic lipids may enhance cellular uptake and endosomal escape. Surface PEGylation reduces nonspecific protein adsorption and prolongs half-life in circulation. Such tunability allows liposomes to function as efficient delivery platforms in cancer vaccine development and immunotherapy (Cao et al., 2015; Li et al., 2014; Nguyen et al., 2016; Sercombe et al., 2015). Both hydrophilic and lipophilic antigens can be effectively loaded into liposomes, which also facilitate the delivery of small molecules to lymph nodes (Detienne et al., 2016).

One notable lipid nanoparticle is Lipoplex, designed by BioNTech. Lipoplex encodes tumor antigen RNA and can elicit robust effector and memory T cell responses. It stimulates both innate and adaptive immune responses, mimicking an antiviral response, and can reject progressive tumors through the action of interferon-alpha (IFNα) (Kranz et al., 2016).

Recent advancements in LNP formulations have significantly enhanced the delivery of nucleic acid-based cancer vaccines, particularly mRNA platforms. Clinically validated LNPs—such as those used in SARS-CoV-2 mRNA vaccines—are composed of ionizable lipids, cholesterol, helper phospholipids, and polyethylene glycol (PEG)-lipid conjugates, which together facilitate endosomal escape, structural stability, and reduced immunogenicity. Optimization of lipid composition, charge ratio, and particle size has shown improved delivery efficiency and tolerability in both preclinical and clinical settings (Buschmann et al., 2021; Hou et al., 2021).

For example, ionizable lipids like SM-102 and ALC-0315 have been used in FDA-approved mRNA vaccines and have demonstrated efficient intracellular delivery and immunogenicity with acceptable safety profiles. In cancer trials, personalized mRNA vaccines using similar LNPs have shown promising tumor-specific immune responses. However, LNPs also present challenges, including off-target delivery, accumulation in the liver and spleen, immune activation, and variability in mRNA release kinetics (Hassett et al., 2019). These limitations emphasize the need for next-generation LNP platforms with enhanced tumor targeting and controlled release characteristics for lung cancer applications.

The liposome-based vaccine BNT111 targets four tumor-associated antigens (MAGE-A3, NY-ESO-1, tyrosinase, and TPTE). Studied in patients with unresectable melanoma, it showed comparable CD4^+^, CD8^+^, and CTL responses to T cell therapy when used alone or with PD-1 checkpoint inhibitors (Sahin et al., 2020).

A notable example of a nanoparticle delivery system for lung cancer is the *in vitro* and animal study by Tang et al., which developed mRNA nanoparticles (NPs) that utilize inhalation as the administration method. These optimized dual-targeted mRNA NPs demonstrated selective accumulation in lung tumor cells and inflammatory macrophages following inhalation, enabling efficient expression of therapeutic proteins such as the tumor suppressor p53. This strategy achieved effective lung tissue transfection *in vivo*, providing strong proof-of-concept for the design and application of dual-targeted mRNA NPs. These advancements underscore the potential of mRNA NP-based inhaled therapies and vaccines for treating lung-related diseases (Tang et al., 2023).

### 4.2 Electroporation-based delivery

Electroporation is employed primarily when DNA vaccines need to be effectively taken up by APCs, which play a crucial role in initiating immune responses. It involves delivering small electrical pulses that create temporary pores in the cell membrane. This process temporarily disrupts the lipid bilayer, allowing plasmid DNA to enter the cell more efficiently than conventional methods (Becker and Kuznetsov, 2007; Roos et al., 2009). Remarkably, the most significant aspect is the substantial enhancement of immune responses triggered by electroporation, which can be as much as 100 times greater than that achieved with traditional injection methods (van Drunen Littel-van den Hurk and Hannaman, 2010).

One of the key advantages of electroporation is its ability to induce an immune response (Cao et al., 2025). The tissue disruption caused by electrical pulses not only facilitates DNA entry but also triggers the release of pro-inflammatory cytokines. These cytokines help recruit and activate various immune cells, thereby amplifying the immune response and providing an additional adjuvant effect. This is particularly beneficial for DNA vaccines, as it enhances their efficacy by promoting a stronger and more durable immune reaction (van Drunen Littel-van den Hurk and Hannaman, 2010; Chiarella et al., 2008).

Despite its advantages, electroporation does come with certain drawbacks. A significant concern is the pain and discomfort that may arise from the procedure, particularly when it is used as a delivery system for vaccines. The electrical pulses can cause localized tissue damage, leading to a sensation of pain that may deter individuals from participating in vaccination programs. Consequently, this makes electroporation less suitable for large-scale vaccination efforts, where patient comfort and efficient vaccine administration are paramount (Paston et al., 2021). Therefore, continued research may enhance its feasibility for widespread vaccination strategies.

A notable advancement in lung cancer immunotherapy is the animal study by Riccardo et al., which employed DNA electroporation as a delivery method for plasmid-based vaccines targeting the ROS1 oncogene. This innovative approach involved the intramuscular electroporation of plasmids encoding both mouse and human ROS1 in transgenic mice with K-Ras-driven lung adenocarcinomas. The study provides compelling evidence for the efficacy of electroporation in facilitating the delivery of therapeutic DNA, highlighting its potential as a transformative strategy for developing effective vaccines against lung cancer and other malignancies (Riccardo et al., 2020).

### 4.3 Gene gun-based delivery

Another promising technique for vaccine delivery and immunotherapy is the gene gun-based delivery system. This innovative method involves using plasmid DNA coated with heavy metals, most commonly gold. During the procedure, APCs at the injection site are bombarded with these plasmid-coated particles. One of the significant advantages of this technique is its efficiency in reducing the amount of DNA required for effective vaccination. Studies have demonstrated that the gene gun method can decrease the necessary DNA dosage by a remarkable factor of 100 to 1,000 compared to traditional delivery methods (Nguyen-Hoai et al., 2022).

### 4.4 Extracellular vesicle-based delivery

One promising method for delivering cancer vaccines or immunotherapies is the utilization of Extracellular vesicles (EVs). These vesicles are lipid membrane-enclosed structures, typically ranging in size from nanometers to micrometers, and are secreted by a wide variety of living cells. EVs play a crucial role in intercellular communication and can carry a diverse array of biological molecules, including proteins, lipids, and nucleic acids (Wiklander et al., 2019; Bhatta et al., 2025).

EVs used in lung cancer vaccine development are primarily derived from DCs, tumor cells, and mesenchymal stem cells (MSCs). DC-derived EVs (also known as dexosomes) can carry peptide–MHC complexes and co-stimulatory molecules, making them promising tools for T cell activation. Tumor-derived EVs can deliver tumor antigens and immunomodulatory factors, contributing to cancer vaccine design. MSC-derived EVs have also been explored due to their biocompatibility and immunomodulatory properties (Bhat et al., 2024; Pitt et al., 2016).

There are three main types of EVs, each distinguished by their size and origin: exosomes, microvesicles, and apoptotic bodies (Yang et al., 2021). These vesicles have gained significant attention in the field of cancer therapy due to their potential to deliver therapeutic agents directly to target cells, thereby enhancing treatment efficacy and reducing side effects (Yang et al., 2021).

For instance, EVs derived from fibroblast-like mesenchymal stem cells can be engineered to carry specific types of RNA, such as small interfering RNA (siRNA) and short hairpin RNA (shRNA). These molecules can target oncogenic KRAS gene involved in cancer progression, making them particularly valuable in the treatment of various cancers, including lung cancer (Kamerkar et al., 2017).

EVs offer numerous advantages as a delivery platform. They possess the ability to navigate natural biological barriers, exhibit inherent targeting properties that direct them to specific cells, and demonstrate remarkable stability during circulation in the bloodstream. These characteristics make EVs an effective medium for delivering therapeutic agents in various medical applications (Ma et al., 2020).

One notable example of a vaccine utilizing this delivery system is the work by Morse et al., which investigates the potential of exosomes derived from autologous dendritic cells (DEX) as an innovative approach to cancer immunotherapy, specifically for patients with NSCLC. This Phase I study focused on HLA A2+ patients with advanced NSCLC who received DEX loaded with MAGE tumor antigens. The findings revealed that DEX therapy was well-tolerated, with only minimal adverse effects reported. Importantly, several patients demonstrated significant durations of disease stability following treatment, suggesting DEX’s ability to activate immune responses against tumors. Overall, this study underscores the feasibility of EV based delivery system as a novel immunotherapeutic strategy for managing advanced NSCLC (Morse et al., 2005).

Despite their potential, EV-based delivery systems face several limitations. The production and isolation of EVs at a clinical scale remain technically demanding due to the lack of standardized, scalable purification methods. Batch-to-batch variability in EV composition and function poses a challenge to consistency and regulatory approval. Moreover, native EVs exhibit limited intrinsic targeting capability, often leading to accumulation in off-target organs such as the liver or spleen following systemic administration (Lener et al., 2015; Wiklander et al., 2015). Overcoming these hurdles through engineering, surface modification, or standardized manufacturing protocols is essential for their clinical translation in lung cancer immunotherapy.

### 4.5 Self-assembling peptides-based delivery

The self-assembling peptide (SAP)-based method is an innovative approach to delivering vaccines and immunotherapies. This method is notable for its resistance to variations in pH, solvent co-assembly molecules, temperature, and ionic strength (Cui et al., 2010; Mandal et al., 2014). Compared to other delivery systems, like nanoparticles, it offers several significant advantages, including high drug loading capacity, low drug leakage rates, biodegradability, and enhanced permeability to target cell membranes. The size of these delivery systems is critical; smaller sizes (20–200 nm) tend to improve immunogenicity (Foged, 2011; Irvine et al., 2013; Xiang et al., 2006).

One prominent system is called “Glycosaminoglycan (GAG)-Binding Enhanced Transduction” (GET), which aims to enhance DNA transfer in lung gene therapy and bone regeneration. This system utilizes cell-penetrating peptides (CPPs) that facilitate cellular entry, targeting sequences for heparan sulfate, endosomal escape peptides to avoid degradation within endosomes, and stabilizing PEG to prevent aggregation. The tripeptide complex can encapsulate DNA into nanoparticles, allowing for intramuscular injection (Abu-Awwad et al., 2017; Dixon et al., 2016; Markides et al., 2019; Osman et al., 2018; Raftery et al., 2019; Spiliotopoulos et al., 2019; Thiagarajan et al., 2017).

This tripeptide formulation has shown remarkable success in DNA delivery, particularly in lung and brain applications, and holds significant potential for vaccine delivery. In summary, self-assembling peptides can be strategically designed to equip vaccines with the necessary properties for efficient targeting and delivery to specific cells (Paston et al., 2021).

SAP-based vaccines show promise due to their biocompatibility but face limitations like weak immunogenicity, poor tissue penetration, and rapid enzymatic degradation (Kim et al., 2019; Malonis et al., 2020). Strategies such as covalent stabilization, adjuvant incorporation, and cell-penetrating peptide design have been proposed to enhance their efficacy (Li et al., 2019; Skwarczynski and Toth, 2016). Further research is needed to improve immune activation and ensure consistent performance in clinical applications (Chen et al., 2017).

### 4.6 Hydrogel-based delivery

Another approach to delivering vaccines or immunotherapy is through hydrogel-based delivery systems (Liu C. et al., 2023). The conventional approach to delivering small drug molecules typically involves dissolving them in a hydrogel, which can lead to suboptimal drug retention and insufficient tumor penetration. In one notable study, researchers used a nanocomposite hydrogel, approximately 6 nm in size, to deliver oxaliplatin in a breast cancer model. The results demonstrated that this nanocomposite hydrogel significantly inhibited tumor growth and metastasis by enhancing the retention and infiltration of anti-cancer agents within the tumor microenvironment (Luo et al., 2022).

By incorporating nanoparticles into this injectable hydrogel formulation, sustained immune activation was achieved, proving to be more effective at inhibiting cancer cell proliferation than conventional intravenous (I.V.) or intraperitoneal (I.P.) injections (Nkanga and Steinmetz, 2022). The use of hydrogels for *in situ* delivery presents numerous benefits, including ease of administration, increased local concentration of therapeutic agents, and prolonged retention times, all of which contribute to effectively preventing tumor recurrence (Wei et al., 2020).

### 4.7 CHP-based delivery

Self-assembled polysaccharide nanogels made from cholesteryl group-modified pullulan (CHP) serve as effective antigen delivery systems for cancer immunotherapy by modulating tumor-associated macrophages (TAMs) (Muraoka et al., 2022). For instance, the cancer-testis antigen NY-ESO-1, which is expressed in various cancers, including lung cancer, has been successfully delivered using CHP in cancer vaccines (Thomas et al., 2018; Kag et al., 2013). This approach offers targeted delivery and enhances the immune responses (Schjetne et al., 2003).

### 4.8 Cell-based delivery

This delivery technique is employed for cell transfer in various therapeutic applications, including T-cell immunotherapy, such as transfusion therapy or tumor-infiltrating lymphocyte (TIL) therapy. In these methods, T-cells harvested from the tumor microenvironment are modified and reinfused into the patient (Rosenberg et al., 2008). Furthermore, this technique is utilized in vaccine development, particularly through DC pulsing, where cells are administered *via* a cell-based platform and injected into the patient to enhance the immune response (Gu et al., 2020).

Beyond the delivery systems already mentioned, researchers are exploring other innovative techniques, like ultrasound, magnetofection, and microbubble-based delivery to enhance the effectiveness of cancer vaccines. These emerging methods, alongside established systems such as nanoparticles, electroporation, and hydrogels, present promising solutions to the current challenges in cancer vaccine delivery. As research progresses, the combination of these diverse approaches is expected to significantly boost the therapeutic potential of cancer vaccines.

## 5 Clinical trials of lung cancer vaccines

Given the urgent need for effective treatments for lung cancer, numerous clinical trials worldwide have focused on developing lung cancer vaccines.

**FIGURE 5:**
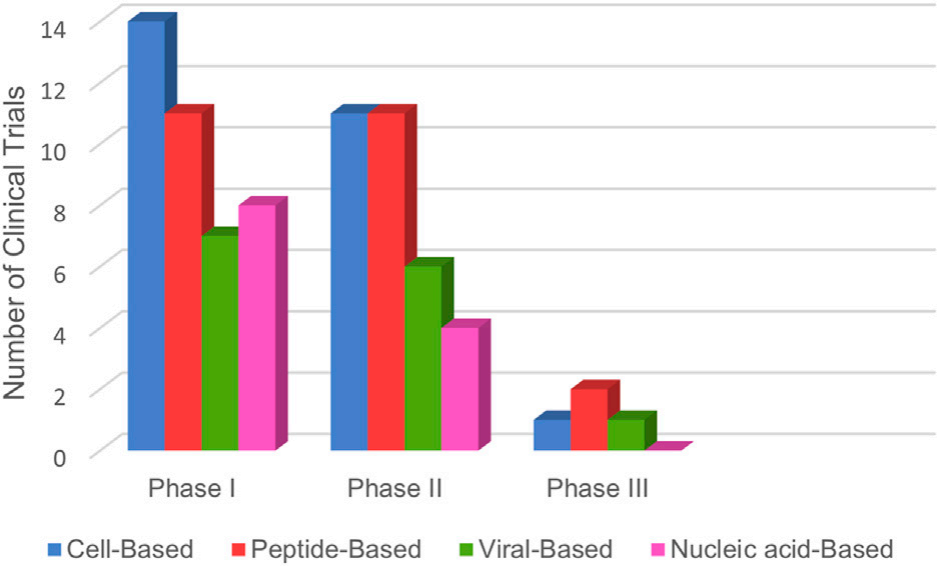
Lung cancer vaccines in clinical trials: The bar graph illustrates the global distribution of therapeutic cancer vaccines for lung cancer, categorized by clinical trial phases. Data was sourced from www.ClinicalTrials.gov, reflecting information up to March 2025.

Upon reviewing Tables 1–4 and Figure 5, it is evident that comprehensive phase III studies demonstrating the full benefits of these vaccines are still lacking. However, laboratory experiments have shown promising responses from CD4^+^ and CD8^+^ T cells against tumor antigens (Palata et al., 2020). Most research has focused on patients with metastatic and advanced NSCLC, with a notable absence of trials involving patients in earlier disease stages. Current studies predominantly target cell-based or peptide-based vaccines, while viral-based and nucleic acid-based options remain less explored, likely due to their emerging status.

In summary, the data indicate that most vaccine studies are still in the early phases of development, highlighting the need for extensive research to advance these promising therapies further.

### 5.1 Cell-based clinical trials of lung cancer vaccines

Cell-based vaccines represent a significant category in the development of lung cancer immunotherapies. In this section, we review four pivotal clinical trials related to cell-based lung cancer vaccination, with a comprehensive summary of selected studies presented in Table 1.

An open-label, phase I pilot study conducted by Hirschowitz EA et al. investigated the effects of a vaccine named 1650-G, which incorporates a tumor-associated antigen DC vaccine alongside GM-CSF. The trial included 11 patients diagnosed with stage I to IIIA NSCLC, all of whom also received an orally administered yeast-derived beta-glucan drug. The primary objective was to evaluate the immune system’s response and T-cell activity following vaccination. Results indicated that the vaccine was safe, with 6 out of 11 participants exhibiting immunological responses (Hirschowitz et al., 2011).

A phase I/II study examined the safety and efficacy of the DC vaccine, known as DCVAC/LuCa, in a cohort of 105 patients with stage IV NSCLC. The research aimed to assess the vaccine in conjunction with chemotherapy agents’ carboplatin and paclitaxel, as well as immunomodulators like interferon-α2b and hydroxychloroquine. Furthermore, it analyzed outcomes from administering DCVAC/LuCa alongside chemotherapy alone. Conclusively, the study found that the combination therapy extended overall survival and was well tolerated, with minimal side effects reported (Zemanova et al., 2021).

A randomized, dose-variable, phase II clinical trial was conducted by Nemunaitis J et al. on 75 patients with stage IIIA, IIIB, or IV NSCLC. The trial involved administering the vaccine Lucanix (belagenpumatucel-L), which consists of allogeneic NSCLC cells. The study focused on evaluating the safety and efficacy of the vaccine, ultimately finding no notable side effects. Additionally, it reported that survival rates correlated with dose escalation, alongside elevated levels of cytokines such as IFN‐γ, IL4, and IL-6 in the participants (Nemunaitis et al., 2006).

An international, multicenter, randomized, double-blind, placebo-controlled phase III study enrolled 270 patients receiving belagenpumatucel-L and 262 control patients. The primary aim was to assess the effectiveness of vaccine therapy compared to a placebo in treating participants. The study concluded with findings indicating that the vaccine was well tolerated, exhibiting no safety concerns. Notably, an improvement in survival was observed for patients who had completed 12 weeks of chemotherapy and received initial radiation (Giaccone et al., 2015).

### 5.2 Peptide-based clinical trials of lung cancer vaccines

Another promising avenue in lung cancer treatment is the development of peptide-based vaccines. In this section, we analyze four key clinical trials in the field of peptide-based vaccines, with a summary of selected studies provided in Table 2.

**TABLE 2:** Summary of selected peptide-based lung cancer vaccination clinical trials listed on ClinicalTrials.gov.

NCT	Phase	Enrollment	Status	Vaccine component	Antigen type	Cancer type	ROA	Summary
NCT06472245	III	363	Recruiting	HER-2/neu, CEA, MAGE 2, MAGE 3, and p53 synthetic peptide	TAA	HLA-A2 Positive Metastatic NSCLC	SC	Peptidic cancer vaccine OSE2101
NCT04397926	I	20	U/S	Individualized neoantigen peptides vaccine	TSA	EGFR mutant NSCLC	SC	Individualized neoantigen peptides vaccine
NCT04487093	I	20	U/S	Individualized neoantigen peptides vaccine	TSA	EGFR mutant NSCLC	SC	Individualized neoantigen peptides vaccine
NCT04263051	II	111	Active, not recruiting	UCP2 and UCP4 (Telomerase)	TAA	Advanced NSCLC	SC	combining Nivolumab with the CD4Th1-inducing vaccine UCPVax
NCT03623750	I/II	23	Completed	EGFR-TK inhibitor	TAA	EGFR mutant NSCLC	N/A	EGFR tyrosine kinase inhibitor combined with EGF pathway-targeting immunization (EGF-PTI)
NCT02654587	III	219	Completed	HER-2/neu, CEA, MAGE 2, MAGE 3, and p53 synthetic peptide	TAA	HLA-A2 positive NSCLC	SC	OSE2101 (TEDOPI)
NCT01935154	II	221	Completed	TRET peptide	TAA	HLA-A*0201 Positive Patients With TERT Positive Stage IV or Recurrent Stage I-III NSCLC	N/A	TRET peptide
NCT00828009	II	70	Completed	MUC1 peptide	TAA	stage IIIA or stage IIIB NSCLC	SC	combining the BLP25 liposome vaccine with Bevacizumab
NCT01219348	I	15	Completed	IDO peptide	TAA	Stage III-IV NSCLC	SC	IDO Peptid Vaccination in Combination With Immune Stimulating Agent Aldara and the Adjuvant Montanide
NCT00874588	I	6	Completed	URLC10, CDCA1, VEGFR1 and VEGFR2 peptide	TAA	Advanced or Recurrent NSCLC	SC	HLA-A*2402 restricted epitope peptides emulsified with Montanide ISA 51
NCT00509457	N/A	20	Completed	Telomerase peptide	TAA	NSCLC	N/A	GV 1001 Telomerase peptide
NCT00019929	II	120	Completed	p53 peptide-pulsed cultured autologous dendritic cells	TAA	Stage III NSCLC	N/A	Individualized Mutant p53 Peptide-Pulsed Cultured Autologous Dendritic Cells

Abbreviations: NCT, national clinical trial; ROA, route of administration; N/A, not applicableID, intra dermal; NSCLC, Non-Small Cell Lung Cancer; LC, lung cancer; PC, pulmonary cancer; N/A, not applicable; SC, subcutaneous; TSA, Tumor-Specific Antigen; TAA, Tumor-Associated Antigen; EGFR-TK, epidermal growth factor receptor tyrosine kinase; CEA, carcinoembryonic antigen; MAGE, melanoma antigen family; HLA, human leukocyte antigen; TRET, telomerase reverse transcriptase; MUC1, Mucin 1; IDO, Indoleamine 2,3-Dioxygenase; URLC10, Upregulated Lung Cancer 10; CDCA1, Cell Division Cycle Associated 1; VEGFR, vascular endothelial growth factor receptor; ISA, immune stimulatory agent.

A Phase I study involved 15 HLA-A2-positive patients with stage III or IV NSCLC who were in the disease stabilization phase following chemotherapy. These patients received IDO peptide vaccine. The study aimed to assess the vaccine’s safety and efficacy, and it reached its final stage without observing severe toxicity. The vaccine was well tolerated, leading to a significant increase in overall survival compared to HLA-A2-negative patients. Additionally, a notable reduction in regulatory T cells was observed among vaccinated patients (Andersen, 2012; Iversen et al., 2014).

Suzuki H et al. conducted a Phase I study that evaluated a peptide-based vaccine designed to assess safety, immunogenicity, and clinical response in patients with advanced NSCLC. The vaccine consisted of two combinations of four HLA-A24-restricted peptides, including two derived from VEGF receptors 1 and 2, one from Regulated Long Cancer 10 (LY6K), and another from TTK protein kinase (CDCA1). Administered subcutaneously with montanide ISA-51 adjuvant, the study concluded with results indicating the vaccine was well tolerated, with no significant adverse events aside from injection site reactions. A specific T-cell response to at least one peptide was noted in 13 out of 15 patients, with 47% experiencing disease stability for a minimum of 2 months (Suzuki et al., 2013).

A Phase II double-blind randomized trial focused on HLA-A*201-positive patients with metastatic NSCLC expressing telomerase reverse transcriptase (TERT) who did not show improvement after platinum-based chemotherapy. Patients were randomized into two groups: one receiving the VX-001 peptide vaccine, which elicits CD8 positive T cell responses against TERT, and the other receiving a placebo. Of the 190 participants, 89 received the vaccine while 101 were in the placebo group. The study concluded without meeting its primary endpoint, revealing a median overall survival of 11.3 months for the placebo group compared to 14.3 months for vaccinated patients, without statistically significant differences. Notably, 29.2% of vaccinated patients exhibited a long-lasting response to TERT, correlating with improved overall survival (Gridelli et al., 2020).

A two-stage open-label Phase III study conducted by Besse B et al. assessed the efficacy and safety of the OSE2101 vaccine, also known as Tedopi, which contains synthetic proteins HER2/neu, MAGE2, MAGE3, and p53. The study compared the vaccine against standard care, which included chemotherapy, in HLA-A2-positive patients with advanced NSCLC who had previously failed chemotherapy and immune checkpoint blockers. Participants were divided into two groups: one receiving the OSE2101 vaccine and the other receiving standard care with docetaxel or pemetrexed. In total, 219 patients participated, with 139 receiving the vaccine and 80 receiving standard care. The study concluded with findings indicating that the vaccine improved survival rates in patients with secondary resistance to immunotherapy compared to chemotherapy, demonstrating better safety outcomes (Besse et al., 2023).

### 5.3 Viral-based clinical trials of lung cancer vaccines

Virus-based vaccines are an innovative lung cancer immunotherapy platform, using viral vectors to deliver genetic material into human cells (Anderson and Schneider, 2007). Research focused on virus-based vaccines in lung cancer has primarily aimed at inducing robust anti-tumor immune responses (Truong and Yoo, 2022). In this section, we evaluate two key clinical trials related to virus-based vaccines, with a summary of selective studies presented in Table 3.

**TABLE 3:** Summary of selected Viral-based lung cancer vaccination clinical trials listed on ClinicalTrials.gov.

NCT	Phase	Enrollment	Status	Vaccine component	Antigen type	Cancer type	ROA	Summary
NCT02840994	I	24	Completed	MUC-1, CEA	TAA	NSCLC	SC	This trial combined CV301, a poxviral-based vaccine, with nivolumab in advanced NSCLC.
NCT04990479	I	34	Active	Adenovirus-Encoded Personalized Vaccine	TSA	NSCLC	IM	This trial evaluates the safety, tolerability, and anti-tumor activity of Nous-PEV combined with pembrolizumab in patients with unresectable stage III/IV cutaneous melanoma and stage IV NSCLC.
NCT03353675	II	44	Completed	MUC1 IL2	TAA	NSCLC	SC	In this trial, researchers evaluated the combination of TG4010 (modified vaccinia of Ankara) with first-line chemotherapy and nivolumab in patients with advanced NSCLC.
NCT02879760	I/II	16	Completed	Maraba Expressing MAGE-A3 (MG1-MAGEA3), with Adenovirus Vaccine Expressing MAGE-A3	TAA	NSCLC	IM/IV	This trial evaluates the safety and efficacy of combining two investigational therapies—Ad-MAGEA3 and MG1-MAGEA3—with pembrolizumab in patients with NSCLC who have previously undergone standard treatment.
NCT01997190	I	19	Completed	Thymidine Kinase adenoviral vaccine	TAA	NSCLC, SCLC	IP	This trial evaluates the safety of intrapleural AdV-tk therapy in patients with malignant pleural effusion (MPE), with secondary objectives of assessing clinical efficacy and biological activity.
NCT00091039	N/A	10	Completed	CEA-TRICOM and fowlpox vaccine	TAA	LC	SC	This clinical trial is studying how well vaccine therapy together with Paclitaxel, Carboplatin, and radiation therapy works in treating patients with stage III NSCLC that cannot be removed with surgery.

Abbreviations, NCT, national clinical trial; ROA, route of administration; N/A, not applicable; TAA, Tumor-Associated Antigen; TSA, Tumor-Specific Antigen; NSCLC, Non-Small Cell Lung Cancer; MAGE, melanoma antigen family; IM, intramuscular; IP, intraperitoneal; ; MUC1, Mucin 1; IL2, Interleukin-2; IT, intratumoral; SC, subcutaneous; IV, intravenous; LC, lung cancer; CEA, carcinoembryonic antigen.

A Phase I dose-escalation trial was conducted in conjunction with chemotherapy for patients with malignant pleural effusion (MPE). In this study, an adenovirus-based vector vaccine expressing thymidine kinase (adV-tk) was administered through intrapleural injection. Following each adV-tk injection, the anti-herpetic prodrug valacyclovir was given orally at a fixed dose for 14 days. The trial involved 19 participants and concluded without any dose-limiting toxicities. Only three patients experienced transient cytokine release syndrome, with one patient also developing hypotension, briefly treated with dopamine. Among the four patients with NSCLC included in the trial, three exhibited prolonged disease stabilization, with one surviving for 29 months after the injection and 3.6 years post-diagnosis. The vaccine demonstrated safety and tolerability in MPE patients undergoing chemotherapy (Aggarwal et al., 2018).

A Phase II/III study conducted by Quoix E et al. evaluated the efficacy of the TG4010 vaccine in combination with chemotherapy *versus* chemotherapy alone in patients with stage IIIb and IV NSCLC. The TG4010 vaccine utilizes a modified vaccinia Ankara carrier that expresses MUC1 and IL2, aimed at inducing an immune response against cancer. A total of 148 patients were enrolled, with 74 receiving the vaccine alongside chemotherapy using cisplatin and gemcitabine, while the remaining 74 served as a control group receiving the same chemotherapy. The study concluded that progression-free survival (PFS) was 43.2% in the vaccine plus chemotherapy group compared to 35.1% in the chemotherapy-only group, although this difference was not statistically significant. Regarding severe adverse events (Grade 3 and 4), only two side effects—anorexia and pleural effusion—exhibited a significant difference, with the chemotherapy group experiencing more incidents. Overall, the study indicated that the TG4010 vaccine enhances the effects of chemotherapy in patients with advanced-stage NSCLC (Quoix et al., 2011).

### 5.4 Nucleic acid-based clinical trials of lung cancer vaccines

Nucleic acid-based vaccines deliver genetic material, such as DNA and RNA, directly into the body. In this section, we examine two key clinical trials in the field of nucleic acid-based vaccines, with a summary of selected studies provided in Table 4.

**TABLE 4:** Summary of selected Nucleic acid-based lung cancer vaccination clinical trials listed on ClinicalTrials.gov.

NCT	Phase	Enrollment	Status	Vaccine component	Antigen type	Cancer type	ROA	Summary
NCT05557591	II	100	Recruiting	Non-Mutated tumor antigens mRNA vaccine	TAA	NSCLC	N/A	This trial compares the combination of Cemiplimab with BNT116 (FixVac Lung) to Cemiplimab monotherapy as a first-line treatment for patients with advanced NSCLC.
NCT05142189	I	130	Recruiting	Non-Mutated tumor antigens mRNA vaccine	TAA	NSCLC	IV	In this trial, researchers evaluate the safety, tolerability, and preliminary efficacy of BNT116 (FixVac Lung) alone and in combinations for patients with advanced NSCLC.
NCT03908671	Pilot	24	Active	Neoantigen personalized mRNA vaccine	TSA	NSCLC, EC	SC	This pilot study evaluates the safety, tolerability, and effectiveness of a personalized mRNA tumor vaccine encoding neoantigens in patients with advanced esophageal cancer and NSCLC.
NCT03164772	I/II	61	Completed	NY-ESO-1 MAGE-C1 MAGE-C2 Survivin 5 T4 MUC1 mRNA vaccine	TAA	NSCLC	ID	This study evaluates the safety and preliminary efficacy of adding vaccine therapy to one or two checkpoint inhibitors in patients with NSCLC.
NCT03313778	I	242	Recruiting	Neoantigen personalized mRNA vaccine	TSA	Solid tumors, LC	IM	This trial evaluates the safety, tolerability, and immune response of mRNA-4157, a personalized cancer vaccine, both as a monotherapy and in combination with pembrolizumab.
NCT00923312	I/II	46	Completed	NY-ESO-1, MAGE-C1/CT7, Survivin, Trophoblast glycoprotein mRNA vaccine	TAA	NSCLC	ID	This trial assesses the safety and efficacy of an RNActive®-derived cancer vaccine designed for patients with stage IIIB/IV NSCLC.
NCT00250419	I	26	Completed	HER-2 and CEA mRNA vaccine	TAA	Solid tumor, NSCLC	IM	In this trial, researchers are evaluating the safety, tolerability, and immunogenicity of an experimental cancer vaccine called V-930. The vaccine targets cancers expressing HER-2 and/or CEA.
NCT05242965	II	40	Recruiting	CDH3 CD105 YB-1 MDM2 SOX2 (DNA)	TAA	NSCLC	ID	This trial evaluates the efficacy of the polyepitope plasmid DNA vaccine (STEMVAC) in reducing tumor size in patients with stage IV NSCLC.
NCT06928922	I	22	Recruiting	melanoma-associated tumor antigens	TAA	LC	inhalation	This study is a dose-escalation trial designed to evaluate the safety, tolerability, and preliminary efficacy of BMD006.
NCT06735508	I	40	Not yet recruiting	Neoantigen	TSA	NSCLC	N/A	This study aims to evaluate the safety, immunogenicity, and preliminary efficacy of a personalized neoantigen mRNA vaccine in combination with adebelimab as adjuvant treatment.

Abbreviations: NCT, national clinical trial; ROA, route of administration; TAA, Tumor-Associated Antigen; TSA, Tumor-Specific Antigen; NSCLC, Non-Small Cell Lung Cancer; N/A, not applicable; EC, esophageal cancer; SC, subcutaneous; ID, intradermal; IM, intramuscular; NY-ESO-1:New York esophageal squamous cell carcinoma-1; HER2, Human Epidermal Growth Factor Receptor 2; CEA, carcinoembryonic antigen; MAGE, melanoma antigen family.

A Phase I study conducted by Diaz CM et al. aimed to evaluate the safety and immunogenicity of the V930 vaccine in cancer patients expressing both HER2 and CEA. The V930 vaccine is a DNA vaccine that includes equal amounts of plasmids encoding extracellular and transmembrane HER2, along with a plasmid expressing CEA fused to the B subunit of *Escherichia coli* heat labile toxin. Involving 26 patients, the study has been completed, with results indicating that the vaccine, in conjunction with electroporation, was well tolerated. No significant adverse effects were reported, apart from localized reactions at the injection site. While ELISPOT detected no cell-mediated immune response to CEA and HER2, a significant increase in cell-mediated immunity and antibody titers against the bacterial heat labile toxin was noted (Diaz et al., 2013).

A Phase I clinical trial conducted by Hussain *et al*. Evaluated the safety and efficacy of BNT116, an mRNA vaccine targeting NSCLC. Utilizing technology similar to COVID-19 vaccines, BNT116 encodes tumor antigens to stimulate cytotoxic T cells against cancer cells. Early results indicate a favorable safety profile and promising antitumor activity. Ongoing studies are assessing its use in combination with checkpoint inhibitors. Despite challenges like mRNA instability, advances in lipid nanoparticle delivery are improving vaccine performance, supporting the potential of mRNA platforms in personalized cancer therapy (Hussain et al., 2025).

A Phase I/II open-label, uncontrolled, international clinical trial involved 46 patients with stage IIIb and IV NSCLC. This study aimed to assess the safety and efficacy of the CV9201 vaccine, which contains mRNA encoding NY-ESO-1, MAGE-C1/CT7, Survivin, and trophoblast glycoprotein. The study has concluded, revealing that the vaccine was well tolerated, with manageable side effects reported. In terms of efficacy, the vaccine successfully induced immune responses in patients, highlighting the potential of mRNA vaccines to activate the immune system against tumors (Fotin-Mleczek et al., 2011).

## 6 Limitations of cancer vaccines

Therapeutic cancer vaccines seek to create a long-lasting immunological memory in the body against tumor cells, resulting in successful tumor regression while limiting non-specific or harmful reactions (Buonaguro and Tagliamonte, 2020). Despite detecting an increase in anti-tumor effector cells following vaccination, clinical trials across various malignancies, including lung cancer, have shown only limited benefits in small-scale populations, with results remaining controversial. Therapeutic cancer vaccines face four primary challenges: immunosuppressive tumor microenvironment, low immunogenicity, established disease burden, and inefficient long-term memory generation (Hollingsworth and Jansen, 2019; Vansteenkiste et al., 2016).

The tumor microenvironment is a complex network that includes various immune components, such as innate and adaptive immune cells, extracellular immune factors, and cell surface molecules (Hegde and Chen, 2020). Immunosuppressive cells within the tumor microenvironment disrupt T cell activation and proliferation by increasing the expression of immunosuppressive receptors such as PD-1 and CTLA-4 and by releasing immunosuppressive cytokines like IL-6, IL-10, TGFβ, and VEGF (Mariathasan et al., 2018; Mazzarella et al., 2019). Furthermore, these immune-suppressive cells have the ability to hinder DC function, resulting in tumor resistance.

Research has demonstrated that lung tumor cells have the ability to generate immunosuppressive substances such as TGF-β, IL-10, cyclooxygenase-2, and prostaglandin E2. As a result, these substances interfere with the ability of DCs to process and present antigens, as well as the anti-tumor activities of T lymphocytes (Gray et al., 2021; Thomas and Massague, 2005). To improve the anti-tumor efficacy of the vaccine, strategies based on the following four perspectives may be able to effectively reverse the suppressive TME: (Bray et al., 2024): depletion of immunosuppressive cells; (Karankar et al., 2025); immune checkpoint inhibition; (Travis et al., 2015); targeting the tumor structure; and (Siegel et al., 2022) enhancing T cell activation or survival signaling (Fan et al., 2023). As previously mentioned, each cancer vaccine platform has its own advantages and disadvantages. The optimal platform would be one that can overcome the immunosuppressive tumor microenvironment while simultaneously activating both the humoral and cellular immune responses.

Another significant challenge in developing an effective cancer vaccine is targeting tumor antigens that may exhibit low immunogenicity within the tumor environment or mutate to evade the immune response (Bowen et al., 2018). Regarding lung cancer, numerous TAAs have been shown to be poorly immunogenic, limiting the effectiveness of cancer vaccinations (Ramlogan-Steel et al., 2014). To ensure the stimulation and development of self-antigen-reactive immune cells, the tolerance should be broken during the development of cancer vaccines by using potent co-stimulators, adjuvants, and repeating the vaccination procedure (Ove and rwijk, 2017). However, as the potency of cancer vaccines increases, there is a higher risk that immune cells will also target tumor antigens in healthy cells, potentially leading to collateral damage and off-target toxicities (Hollingsworth and Jansen, 2019). Additionally, identifying novel TAAs or targeting multiple antigens, along with designing vaccines based on both new and specific antigens and utilizing appropriate delivery platforms, could lead to more effective treatments and enhanced immune responses in lung cancer.

Also, due to variations in immunogenicity among tumor cells, those with high immunogenicity trigger a strong anti-tumor immune response and are typically eradicated by the body. Conversely, tumors that have low immunogenicity can escape the immune system and undergo selective proliferation; a process known as immune selection. The tumor’s immunogenicity gradually diminishes over time due to ongoing selection. Furthermore, low expression of HLA molecules and impaired antigen presentation contribute to tumor immune evasion (Liu et al., 2022).

The study population may be an additional problem. It is evident that the effectiveness of cancer vaccines is reduced by the fact that most clinical trials assess vaccination in patients with advanced or metastatic lung cancer. However, cancer vaccines may demonstrate greater efficacy when administered in cases where the disease burden is low (Ramlogan-Steel et al., 2014).

Despite the previously mentioned challenges, there are additional obstacles. One of the primary limitations at the moment is the absence of validated biomarkers that can guide optimization and predict vaccine efficacy. A precise understanding of which T cell subtypes are crucial for an effective cancer vaccine and how to specifically stimulate them is essential too. Moreover, the overall success of cancer vaccinations can vary significantly between cancer types and individual patients, necessitating personalized approaches. However, creating personalized vaccines with neoantigens unique to each patient is a costly, time-consuming, and technically challenging process.

## 7 Combinations with other therapies

Despite progress in cancer vaccine therapy, these vaccinations alone have not elicited a sufficient response to independently eradicate cancer (Melief et al., 2015; Maeng et al., 2018). Therefore, to address different treatment challenges, specific combined approaches have been developed, considering the specific pathological features of each tumor, various drug resistance mechanisms, and the benefits and drawbacks of different vaccination platforms. According to recent research, treatments that combine previously investigated medications with cancer vaccinations yield much more encouraging outcomes (Maeng et al., 2018; Kantoff et al., 2010). To investigate potential synergies, vaccine combinational strategies involving cytokines, ICIs, radiotherapy, small molecules, endocrine therapy, chemotherapy, and hormonal therapy have been explored.

Recently, there has been active research into vaccine combinations involving checkpoint inhibitors, such as anti-CTLA-4 or anti-PD-1/PD-L1, to enable the vaccine-induced T cells to enter the tumor and function there without being inhibited by ligands like PD-L1 (Gatti-Mays et al., 2017; Parch et al., 2016). This approach aims to improve cancer cell recognition through the vaccine while boosting the immune response by utilizing checkpoint inhibitors (Pardoll, 2012). The underlying mechanism is that cancer vaccines work to enhance T cells’ ability to recognize tumor antigens, but the immune response can be weakened by the tumor’s evasion strategies (Morse et al., 2021). Immune checkpoint inhibitors address this by blocking inhibitory pathways on T cells, effectively “removing the brakes” and boosting their antitumor activity (Igarashi and Sasada, 2020).

The immune system developed to protect against microbiological infections, so for a vaccine to imitate an invading microbe, it needs to be perceived as both foreign and harmful. Both of these signals can be produced by vaccination technologies based on viruses, bacteria, and nucleic acids; however, peptide vaccines lack the necessary “danger” signal. In addition, delivering an antigen without the necessary co-stimulators causes T cell ignorance, anergy, or even T cell deletion (Hailemichael et al., 2013). As a result, significant efforts have been focused on creating potent adjuvants that mimic pathogen- and damage-associated molecules, which are recognized by PRRs, including TLRs. A wide range of adjuvants capable of triggering PRRs have been employed in preclinical cancer vaccine investigations, with some being evaluated in clinical trials (Hailemichael et al., 2013; Kumai et al., 2017; Khong and Overwijk, 2016).

Recent reports have frequently highlighted the importance of combining cancer vaccines with chemotherapy and radiotherapy. Since these therapies are standard treatments for many cancers, the development of most cancer vaccines will involve patients who have already received or are undergoing these treatments. Therefore, it is essential to understand how these therapies interact with cancer vaccines to optimize their efficacy and improve patient outcomes.

Radiotherapy may, when combined with tumor vaccines, work synergistically by triggering the release of cancer cell antigens and activating the immune system, thereby enhancing the effectiveness of cancer vaccines (Liu D. et al., 2023). However, there are drawbacks to this combination strategy. For instance, the ideal timing and dosage of radiotherapy in relation to the vaccine remain unclear, raising the risk of radiotherapy potentially damaging immune cells activated by the vaccine and diminishing the overall treatment effectiveness (Lugade et al., 2005).

Over the past few years, a growing body of research has revealed that the effectiveness of certain traditional chemotherapy drugs depends on immune system modulation in addition to their direct cytostatic and cytotoxic effects (Galluzzi et al., 2015). The potential synergy between cancer vaccines and chemotherapy results from certain chemotherapeutic agents triggering immunogenic cell death, making dying cancer cells more recognizable to the immune system, which may enhance the effectiveness of cancer vaccines (Obeid et al., 2007).

Active research is currently being conducted on combinations of ICIs and chemotherapy; for instance, adding pembrolizumab to standard chemotherapy in patients with non-small cell lung cancer resulted in a significantly longer overall survival (OS) compared to chemotherapy alone (Gandhi et al., 2018). Numerous studies in animal models have demonstrated that chemotherapy can also work effectively in conjunction with cancer vaccines, leading to clinical testing of these combinations (Gatti-Mays et al., 2017). For example, the combination of platinum-based chemotherapy and TG4010, a modified Ankara virus vaccine that expresses MUC-1 and IL-2, was investigated in a phase IIb/III trial involving patients with advanced non-small cell lung cancer (Quoix et al., 2016). When compared to chemotherapy alone, the combination group in this trial exhibited a longer median PFS and more confirmed responses. These results show that chemotherapy can improve the effectiveness of cancer vaccines by modifying immune responses.

However, this approach faces significant challenges, including the difficulty of determining the optimal timing and dosage of chemotherapy in conjunction with cancer vaccines. Additionally, both treatments come with side effects: chemotherapy can cause fatigue, infection, hair loss, and nausea, while cancer vaccines have their own adverse effects (Liu D. et al., 2023).

Evidence indicates that the therapeutic cancer vaccines currently undergoing clinical research are unlikely to significantly influence cancer outcomes as standalone treatments. Numerous combination strategies have been attempted, including checkpoint inhibitors, small molecule inhibitors, radiation therapy, chemotherapy, and vaccination plus cytokines. These studies suggest that the most promising approach for enhancing clinical outcomes is the combination of therapeutic vaccines with immune checkpoint inhibitors.

## 8 Conclusions and future perspectives

Lung cancer, particularly NSCLC, remains a leading cause of cancer deaths, with low survival rates despite advancements in conventional therapies. Immunotherapy, including ICIs and CAR-T therapies, offers promise, but challenges like limited patient response and drug resistance highlight the need for alternative approaches such as cancer vaccines. These vaccines, designed to stimulate the immune system to target tumor cells, show potential in transforming “cold” tumors into “hot” ones. However, their clinical efficacy remains limited by factors like antigen loss and tumor immune evasion. While preliminary results are promising, cancer vaccines still require further development, particularly in combination with other therapies.

Future research should focus on improving antigen selection, with emphasis on tumor-specific antigens and personalized neoantigen vaccines. Advancements in delivery systems, including nanoparticle-based and nucleic acid vaccines, are essential for enhancing vaccine efficacy. Combining vaccines with other treatments like ICIs and chemotherapy offers potential for overcoming immune resistance, but requires optimization of timing and dosage. Lastly, the identification of reliable biomarkers will be critical in personalizing vaccine therapies and improving patient outcomes. Addressing these challenges will help integrate cancer vaccines into standard lung cancer treatments.
